# Space–time distribution of classical scrapie in Italian sheep: assessing the effectiveness of the National Genetic Selection Plan

**DOI:** 10.1186/s13567-025-01677-8

**Published:** 2025-12-06

**Authors:** Rosanna Desiato, Francesco Ingravalle, Silvia Bertolini, Eleonora Aiassa, Paola Barzanti, Maria Gabriella Perrotta, Dolores Catelan, Giuseppe Ru

**Affiliations:** 1 Italian Reference Centre for Animal Transmissible Spongiform Encephalopathies, Istituto Zooprofilattico Sperimentale Piemonte, Liguria e Valle d’Aosta, Turin, Italy; 2 S.C. Servizio Veterinario Area A ASL TO4, Settimo T.se, Turin, Italy; 3 Ex Direzione Generale della Sanità Animale e dei Farmaci Veterinari, Ministero della Salute, Rome, Italy; 4 Unit of Biostatistics, Epidemiology and Public Health, Department of Cardiac, Thoracic, Vascular Sciences and Public Health, University of Padua, Padua, Italy

**Keywords:** Scrapie, surveillance system, prevalence, National Genetic Selection Plan

## Abstract

Scrapie is a contagious neurodegenerative disease of sheep and goats sustained by prions. The aim of this study was to assess the effectiveness of selective interventions in Italy by describing the temporal trends and geographical distribution (a) of scrapie prevalence and (b) of the application of the National Genetic Selection Plan (NGSP). Poisson models were used to study temporal trends of disease prevalence (in terms of prevalence ratios, PR), whereas NGSP genotyping data from rams tested between 2005 and 2022 were used to study the temporal trend and geographical distribution of the percentage of susceptible animals. In addition, change-point regression analysis was used to identify changes in the trend. The trend in prevalence at the national level shows an increase in the period 2005–2015 (PR = 1.09, 95% CI 1.04–1.15), followed by a sharp decline between 2016 and 2022 (PR = 0.88, 95% CI 0.83–0.93). The proportion of susceptible rams shows a decreasing temporal trend, both along 2005–2022 (PR 0.97, 95% CI 0.96–0.98) and in the two periods (respectively, 2005–2015: PR 0.92, 95% CI 0.89–0.96; 2016–2022: PR 0.94, 95% CI 0.89–0.98). Change-point regression analysis identified a nationwide trend shift between 2015 and 2020, with Sardinia showing an earlier change between 2012 and 2015. The analysis of temporal trends showed geographical heterogeneity associated with different control strategies, especially in Sardinia, where the early targeted interventions in 2009 anticipated most of the provisions of the second NGSP.

**Supplementary Information:**

The online version contains supplementary material available at 10.1186/s13567-025-01677-8.

## Introduction

Scrapie is a chronic progressive neurodegenerative disease with a fatal outcome that affects the nervous system of sheep and goats and is sustained by a prion agent [1, 2]. It is a naturally occurring transmissible spongiform encephalopathy (TSE), causing a protein misfolding of the normal prion protein (PrP^C^) into a pathogenic form (PrP^Sc^) that is highly resistant to enzymatic breakdown and accumulates in the cells, eventually leading to neurodegeneration [3]. Other examples of prion diseases include bovine spongiform encephalopathy (BSE) in cattle, chronic wasting disease in cervids (CWD) and Creutzfeldt–Jakob disease (CJD) in humans, with the new variant (vCJD) caused by BSE [4]. Similar to many TSEs, scrapie is characterized by a long incubation period, typically affecting animals between the ages of 2 and 5 years. Affected animals can survive for 1–6 months after the onset of clinical signs [5, 6]. There are two main forms of scrapie, classical scrapie and atypical scrapie. Classical scrapie (CS) is a disease well-known since the 18-century, as it was first described in the UK in 1732 [7]. Since then, classical scrapie has been reported worldwide apart from Australia and New Zealand, so classical scrapie is now considered endemic in many States, also in the European Union. As a transmissible disease, it can spread within a flock, by direct contact of animals and among flocks by trade, bringing economic losses due to decreased production, export loss and increased cost for carcass disposal.

Atypical scrapie (AS) was first detected in Norway in 1998 and is different from classical scrapie in clinical presentation, molecular characteristics and distribution of PrP^Sc^ (that in infected hosts is restricted to central nervous system), genotypes affected and also in epidemiology [8]. Atypical scrapie has been identified in Europe, North America, New Zealand and Australia [9–11]. There is no evidence that AS can be transmitted between animals under natural conditions [12] and it is considered more likely that AS is a non-contagious disease [13]. Risk factors, apart from age and genetic susceptibility, remain largely unknown.

In classical scrapie, the pathological isoform of the prion protein accumulates predominantly in central nervous tissue. However, the widespread distribution within lymphoid tissues [14] significantly contributes to the potential environmental contamination of pastures and farms with placenta as the primary vehicle of transmission. Classical scrapie can be transmitted horizontally among sheep or goats by direct or indirect contact and vertically from sheep to their offspring in the period from parturition to weaning [15]. The infectious material can persist for several years on pastures and in buildings. Milk from clinically affected animals can also transmit disease [16–19].

Classical scrapie is a disease modulated by genetic factors, in particular the amino acids encoded in specific prion protein codons (136, 154 and 171) have an important effect on susceptibility to the agent and incubation periods. Several specific allelic forms have been identified that confer resistance or susceptibility in sheep: ARR (alanine at codon 136, arginine at codon 154, and arginine at codon 171), which confers resistance; VRQ (valine at codon 136, arginine at codon 154, and glutamine at codon 171), associated with high susceptibility; and AHQ, ARH, and ARQ variants, which are also associated with susceptibility although ARQ/ARQ sheep are common in most breeds and account for a significant number of classical scrapie cases, e.g. in Italy [20, 21].

In this work, only classical scrapie (most likely more than one strain circulates in Italy, [22, 23]), has been considered, whereas the atypical scrapie has not been taken into account because of its epidemiological, biochemical and histopathological differences.

Surveillance activities implemented since 1995, based on passive surveillance, showed that scrapie, although it is a rare disease characterized by low prevalence levels, was widely spread in Italy. Regulation (EC) No 999/2001 [24] still in force today provided rules and criteria for the prevention, control and eradication of scrapie in small ruminants, based on active surveillance plan. In accordance with this regulation, the monitoring of ovine and caprine animals for the presence of scrapie is based on the testing of samples taken from different target groups. Specifically, active surveillance relies on rapid tests carried out on a national sample of healthy slaughtered animals and fallen stock aged 18 months and over. Passive surveillance requires confirmatory testing of clinically suspect animals irrespective of age. Over time, culling measures have evolved with the epidemiological scenario, moving from total herd culling in the early 2000 s to derogations slaughtered for human consumption and the retention of scrapie resistant individuals (selective culling). This shift has been enabled by increased understanding of genetic resistance to scrapie and the implementation of genetic selection programs.

The active surveillance programme has remained unchanged for the last 15 years and Italy is required to carry out at least 10 000 rapid tests per category (regularly slaughtered and dead) in each species (sheep and goats over 18 months of age), based on representative samples accounting for season, geographical area and livestock system. The minimum sample size for the monitoring of TSEs (active surveillance) is calculated according to Regulation 999/2001 and is based on the national consistency for fallen stock and on the slaughter volume for healthy slaughtered animals.

As scrapie is a disease with genetic susceptibility, genetic selection is an effective strategy to control and eradicate the disease as there are currently no vaccines or in vivo diagnostic tests [25, 26]. As confirmed in the latest EU summary report on TSE [27], classical scrapie is declining in many European countries in populations subject to breeding for resistance, although it is still present with a high incidence in southern European countries such as Italy [28]. In line with EU guidelines, Italy implemented a National Genetic Selection Plan (NGSP) in 2005 (Decree 17/12/2004) [29], initially annually targeting all rams and ram lambs from high genetic merit flocks on a mandatory basis, while allowing commercial flocks to participate voluntarily. In 2016, the plan was revised and expanded (Decree 25/11/2015) [30] to make participation compulsory for all Italian flocks. In Sardinia, which houses half of Italy’s sheep population, the inclusion of commercial flocks had already been mandated in 2009. Both versions of the NGSP required each Italian region to develop a plan consistent with the national framework. The purpose of the plan was to increase the frequency of the ARR/ARR genotype in the sheep population and thus make animals more resistant to scrapie.

Assessing the effectiveness of this type of intervention, which still requires major efforts by health authorities and active cooperation from breeders, is crucial to achieving the control of the disease.

The study of the spatio-temporal distribution of the disease can provide relevant insights into its epidemiology, determining the role of risk factors influencing its spread and verifying the effectiveness of the NGSP.

This study aims to describe the temporal trends and geographical distribution of (a) the prevalence of scrapie, (b) the application of the NGSP and (c) the change in allele and genotype frequencies in the sheep population following the introduction of the NGSP, using statistical and epidemiological tools to evaluate the effectiveness of current selective interventions.

## Materials and methods

### Study area and small ruminant population

This is a national study based on the Italian TSE surveillance plan and the NGSP. As to December 2024 (ISTAT), in Italy there are 135 702 farms where 6 497 003 sheep and 979 913 goats are bred. Sardinia alone accounts for 40% of the national sheep and goat population.

### Database

Raw data are electronically submitted by each Institute for Zooprophylaxis at national level to the Reference Centre for Animal Encephalopaties (CEA)–Institute for Zooprophylaxis in Turin. The data submitted consist of testing data and case-based data for small ruminants, according to the reporting periods (monthly basis) as described in Chapter B.I of Annex III of the TSE Regulation.

Data have been retrieved from two different databases: the National Surveillance for TSEs (NSTSE) and the NGSP, both maintained by the CEA.

### National TSE surveillance data

NSTSE data on scrapie occurrence were subjected to detailed descriptive epidemiological analysis to identify and describe differences in geographical distribution and temporal trends. The study covers the period from 1 January 2002 to 31 December 2022.

This dataset included data from a total of 569 084 animals reared in a total of 61 288 flocks and tested in the frame of the active surveillance scheme. While passive surveillance is based on the mandatory reporting of any animal presenting clinical sign compatible with scrapie, the active scheme provides on an annual basis the post-mortem testing of a national sample of sheep from two targeted streams i.e. animals slaughtered for human consumption (SHC i.e. healthy slaughtered) and animals not slaughtered for human consumption (NSHC, i.e. fallen stock), both aged 18 months and over. In this study 375 769 SHC sheep and 193 315 NSHC sheep have been considered. Data have been aggregated at macro-region (i.e. aggregation of regions), regional and provincial level.

Macro-region 1 had the highest prevalence, with 89 positive tests out of 39 970 (0.23%). This was closely followed by macro-region 6, which recorded 284 positives out of 138 886 tests (0.20%). Macro-region 4 had the lowest prevalence, with 94 positives out of 152,038 tests (0.06%). The prevalence in the other macro-regions ranged from 0.10% to 0.13% (Table 1).

**Table 1:** **Scrapie surveillance data in sheep from 2002 to 2022**

Macro-region	1	1	2	2	3	3	4	4	5	5	6	6			
Year	*N* positives	*N* tested	*N* positives	*N* tested	*N* positives	*N* tested	*N* positives	*N* tested	*N* positives	*N* tested	*N* positives	*N* tested	Total positives	Total tested	% (positives/tested)
2002	2	978	5	615	9	4618	7	5501	9	6016	2	5160	34	22 888	0.15
2003	3	1266	4	927	9	8553	3	9909	4	6233	4	11 743	27	38 631	0.07
2004	1	792	0	668	3	5336	1	4080	4	2791	3	7363	12	21 030	0.06
2005	0	1106	2	763	8	5383	1	5677	3	2477	6	5481	20	20 887	0.10
2006	3	4386	1	1419	19	13 640	5	13 709	2	8608	13	9567	43	51 329	0.08
2007	6	5672	0	1860	12	22 508	5	21 454	9	16 280	15	13 381	47	81 155	0.06
2008	3	2071	4	2925	10	7462	2	7742	0	2793	17	5094	36	28 087	0.13
2009	4	1147	0	2500	10	4963	8	6059	0	1718	22	5079	44	21 466	0.20
2010	2	950	0	2395	4	4061	3	6010	0	784	31	6701	40	20 901	0.19
2011	5	1375	8	2299	9	4103	13	6382	1	1137	55	6999	91	22 295	0.41
2012	5	1565	3	2219	11	2919	4	5486	6	1536	19	5486	48	19 211	0.25
2013	10	1588	5	2275	8	3859	8	5884	3	1813	15	4347	49	19 766	0.25
2014	7	1618	5	2064	12	5211	5	7316	2	1548	7	2141	38	19 898	0.19
2015	9	1747	5	1911	9	4721	3	5582	12	1677	7	3248	45	18 886	0.24
2016	8	1900	2	2369	9	4812	1	5931	1	1233	3	4196	24	20 441	0.12
2017	2	1912	2	2171	11	4934	12	6773	4	850	16	5089	47	21 729	0.22
2018	3	1980	1	2345	6	5475	2	6346	2	1215	11	6718	25	24 079	0.10
2019	6	2393	0	2824	5	5683	3	6659	1	1199	13	7197	28	25 955	0.11
2020	4	1974	2	2351	5	5890	2	5178	1	1426	5	6270	19	23 089	0.08
2021	5	1926	3	2897	4	5145	5	5436	0	1443	10	9414	27	26 261	0.10
2022	1	1624	0	1850	2	3326	1	4924	1	1164	10	8212	15	21 100	0.07
Total	89	39 970	52	41 647	175	132 602	94	152 038	65	63 941	284	138 886	759	569 084	0.13

Data are presented as the number of positive and tested sheep and their respective proportions by year and macro-region. Macro-regions are: (1) North-West, (2) North-East, (3) Central, (4) Southern Italy, (5) Sicily and (6) Sardinia.

### National genetic selection plan

Susceptibility to scrapie is determined by genotyping the prion protein gene (*PRNP*), in particular the haplotypes at codons 136, 154, 171.

The genotyped ovine population in Italy reveals demographic and geographical patterns similar to the distribution of sheep population. Notably, 76% of the genotyped animals are male, with 83% of these being under 2 years of age. Age distribution data show that 10% of genotyped animals are less than 1 year old. In terms of geography, Sardinia contributes the largest share, accounting for 38% of all genotyped animals and 40% of genotyped males for breeding plan selection, highlighting the region’s importance in sheep breeding activities. Central Italy (macro-region 3) represents 16% of the genotyped sheep population, consistent with the region’s high density of sheep flocks. By contrast, the northern provinces of Trento and Bolzano contribute a smaller proportion, accounting for just 5% of genotyped animals. The dataset included data from a total of 834 996 sheep including 568 522 rams less than 2 years old genotyped in the frame of the NGSP from 2005 to 2022 (Additional file 1). An in-depth analysis of the NGSP data was then carried out focusing on its implementation at the regional level. Geographical and temporal differences were considered looking at: (1) the application of the plan in terms of genotyped animals; (2) the plan results in terms of changes in allele frequencies of disease susceptibility traits in male animals less than 2 years old.

### Diagnostic tests

Samples of medulla oblongata at the level of the brainstem collected by the veterinary officers of the Local Veterinary Units from ovine and caprine animals were sent for laboratory testing and examined by a rapid test to ensure the detection of all known TSE strains in the network of Institutes for Zooprophylaxis (Annex X of Regulation (EC) 999/2001). If the rapid test was inconclusive or positive, the sampled tissues were sent to the CEA laboratory for confirmatory examination by histopathology, immunohistochemistry (IHC), western blot analysis (WB). Western blot and immunohistochemistry involve proteolytic digestion with proteinase K (PK) to detect the PrP^Sc^ fragment (PrP^res^) using specific antibodies. The disease was confirmed if one of these tests, histology, IHC or WB was positive. These tests confirm the diagnosis and can also differentiate between classical and atypical scrapie on the basis of different electrophoretic patterns. The protease-resistant core of PrP^Sc^ (PrP^res^) was typed by discriminatory immunoblotting using the discriminatory WB method of the Istituto Superiore di Sanità (ISS) to distinguish classical scrapie from atypical scrapie.

### Statistical analysis

#### Prevalence data

**Figure 1:**
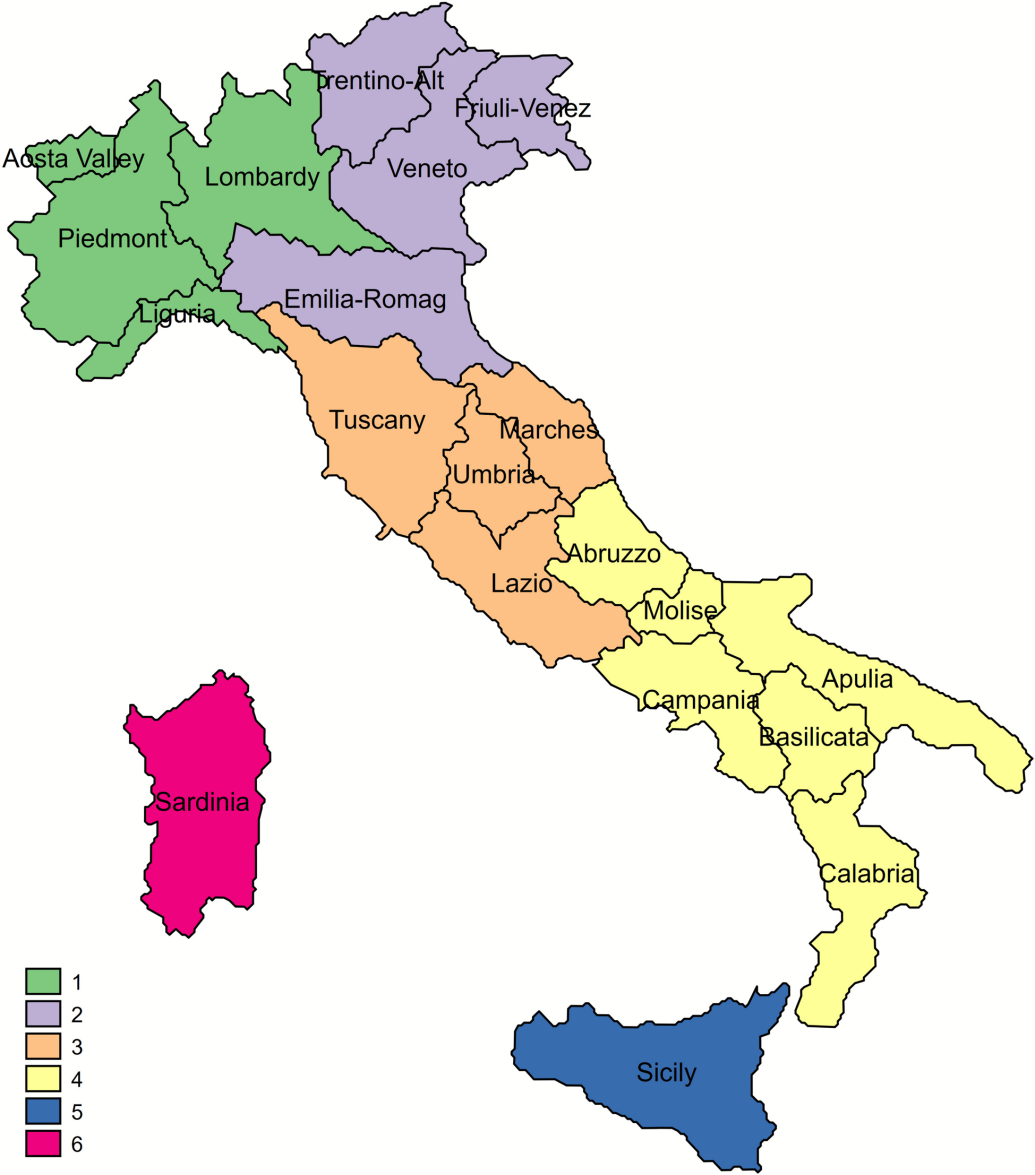
**Classification of Italy according to macro-regions as defined by the Italian National Statistics Institute (ISTAT)**.

Based on the surveillance data, crude prevalence (i.e. the proportion of tested sheep with a positive result out of the total number of tested sheep) and adjusted prevalence at individual level were calculated. Prevalence was adjusted through direct standardization on surveillance stream (SHC and NSHC) and age class (18–47 months versus over 47), based on average incubation period of developing disease). The time course of scrapie adjusted prevalence was assessed along two different periods relating to the enforcement of two successive selection plans: (1) from 2005 to 2014 based on the DM 17/12/2004, when genotyping was mandatory for flocks of high genetic merit; (2) from 2015 to 2022 based on DM 25/11/2015, when genotyping was made mandatory for all flocks (see next section below for details). Moreover, data were aggregated into six macro-regions, according to the geographical classification of the Italian Institute for statistics (ISTAT) to verify any potential differences in prevalence: (1) North-West Italy, including Piedmont, Liguria, Valle d’Aosta and Lombardy, (2) North-East Italy with Trentino, Veneto, Friuli, Emilia-Romagna, (3) Central Italy with Tuscany, Lazio, Umbria, Marche, (4) Southern Italy including Abruzzo, Molise, Puglia, Basilicata, Campania, Calabria, (5) Sicily, (6) Sardinia (Figure 1).

Trends in prevalence have been modelled through regression techniques. Given that the number of events (*n*, cases) was large and the probability of infection (*p*) very small [31], we used the Poisson distribution as a good approximation of the binomial distribution. A Poisson regression model with robust variance was fitted (model 1) where the number of cases was treated as the dependent variable, and year as a continuous independent variable. The number of tests was accounted for as an offset in the model. In addition, the surveillance stream (NSHC versus SHC) and age class, which could potentially affect the probability of detecting the disease, were added to the model as confounding variables.

The prevalence ratio (PR), obtained by exponentiating the beta coefficient associated with the "year" variable, was used to quantify the annual variation in the probability of detection, i.e., the temporal trend for the entire period. In the model, the PR indicates the average annual change in the proportion of cases per animal tested, corresponding to the annual probability disease detection. A PR > 1 indicates an average annual increase in the number of cases per million whereas a PR < 1 indicates an average annual decrease.

To assess variation over time, a change point regression analysis was performed [32], using the *bayesmh* command in Stata to detect the time period of change (expressed as mean, standard deviation, median and credibility interval of the posterior distribution for the change point and the associated Monte Carlo Standard Error, MCSE) in the trend of scrapie prevalence: the analysis has been carried out considering the entire country and then, separately, Sardinia versus the rest of Italy.

#### Genotyping data

The genotyping data obtained from the NGSP have been restricted to rams under 2 years of age, with the focus being on the newborn resulting from the selective intervention. The goal of the plan in both the original and the second implementation (i.e. the periods 2005–2015 and 2016–2022) was to increase the frequency of genetic resistance traits to TSEs in the national sheep population in order to contribute to the eradication of TSEs in sheep and to create ‘low-risk’ TSE flocks. Increasing resistance traits is achieved by creating reservoirs of homozygous resistant rams (with genotype ARR/ARR), which are also useful for restocking infected flocks. In this study, we considered as susceptible all animals with genotypes containing the VRQ allele (e.g., VRQ/VRQ, ARQ/VRQ, ARR/VRQ, AHQ/VRQ), as well as those commonly associated with susceptibility such as ARQ/ARQ, ARQ/AHQ, and AHQ/AHQ.

To assess the effectiveness of the NGSP on the reduction of susceptible animals over time and by macro-region, a second Poisson model (model 2) was fitted using the number of susceptible animals (rams under 2 years-old) as the dependent variable and the year of sampling as the independent variable. The number of genotyped animals was taken into account in the model as offset. The same model was then applied to each macro-region. The proportion ratio (PR) of susceptible animals obtained by exponentiating the beta coefficient associated with the ‘year’ (as a continuous variable) was used as a measurement of the annual variation in the proportion of susceptible rams for the entire period.

The effect of the proportion of susceptible animals on disease prevalence has been quantified through a Poisson mixed regression model (model 3). The dependent variable was the number of cases, taking into account the number of tests as offset. The independent variable was the proportion of susceptible animals, with a 3-year lag between the time of case sampling and the NGSP sampling. This time lag was chosen to account for the time required for genetic selection in rams to influence the population, as it typically takes one to two generations for changes in susceptibility traits to propagate within the population [33]. Thus, the 3-year lag provides a window to observe the potential impact of the NGSP on reducing scrapie prevalence. To account for potential clustering, the Italian province was included as a random effect. The prevalence ratio (PR), obtained by exponentiating the beta coefficient associated with the proportion of susceptible animals, was used as a measure of the change in the probability of detecting scrapie on the basis of the change in the proportion of susceptible animals.

Quartiles were defined for the proportion of susceptible animals and the lowest quartile was compared with all the others (first quartile < 22%, second quartile > 22–33%; third quartile > 33–48%; fourth quartile > 48%). For further analysis, the proportion of susceptible animals was also categorized into a dichotomous variable, comparing the first quartile (0–22%) with all other quartiles combined (> 22%).

To visually compare the recent epidemiological status of scrapie with trends in the proportion of susceptible animals resulting from the NGSP, two maps were produced at the provincial level. For the scrapie situation, focusing on the last 5 years, a Poisson regression model (model 4) was fitted using the number of scrapie cases detected between 2018 and 2022 as the dependent variable and province as the independent variable, with the number of tests included as an offset. Surveillance stream and age group were also added as covariates for adjustment. The resulting province-specific adjusted prevalence rates were used to create a prevalence map. For trends in the proportion of susceptible animals, a separate Poisson regression model (model 5) was fitted for each province. In this model, the number of susceptible animals per year was used as the dependent variable and year as a continuous independent variable, again with the number of tests as an offset. In this case the considered period (i.e. 2016–2022) corresponded to the enforcement of the revised and expanded NGSP which provided for the compulsory participation of both the commercial and the high genetic merit flocks The resulting province-specific proportion ratios (PS) were visualized on a map: provinces with an increasing trend (PS > 1, *p*-value < 0.05) were marked in red; provinces with a decreasing trend (PS <p 1, *p*-value < 0.05) were marked in green; and provinces where the model showed no significant trend (*p*-value > 0.05) were marked in yellow.

#### Software

Stata^®^ Software version 17 [34] was used to create the databases and process the data for the statistical analysis. The Poisson model was fitted using the *poisson* function. Changepoint regression analysis was performed using *bayesmh* command. Stata^®^ Software version 17 was also used to create the thematic maps with the *spmap* package.

## Results

### Prevalence data

**Figure 2:**
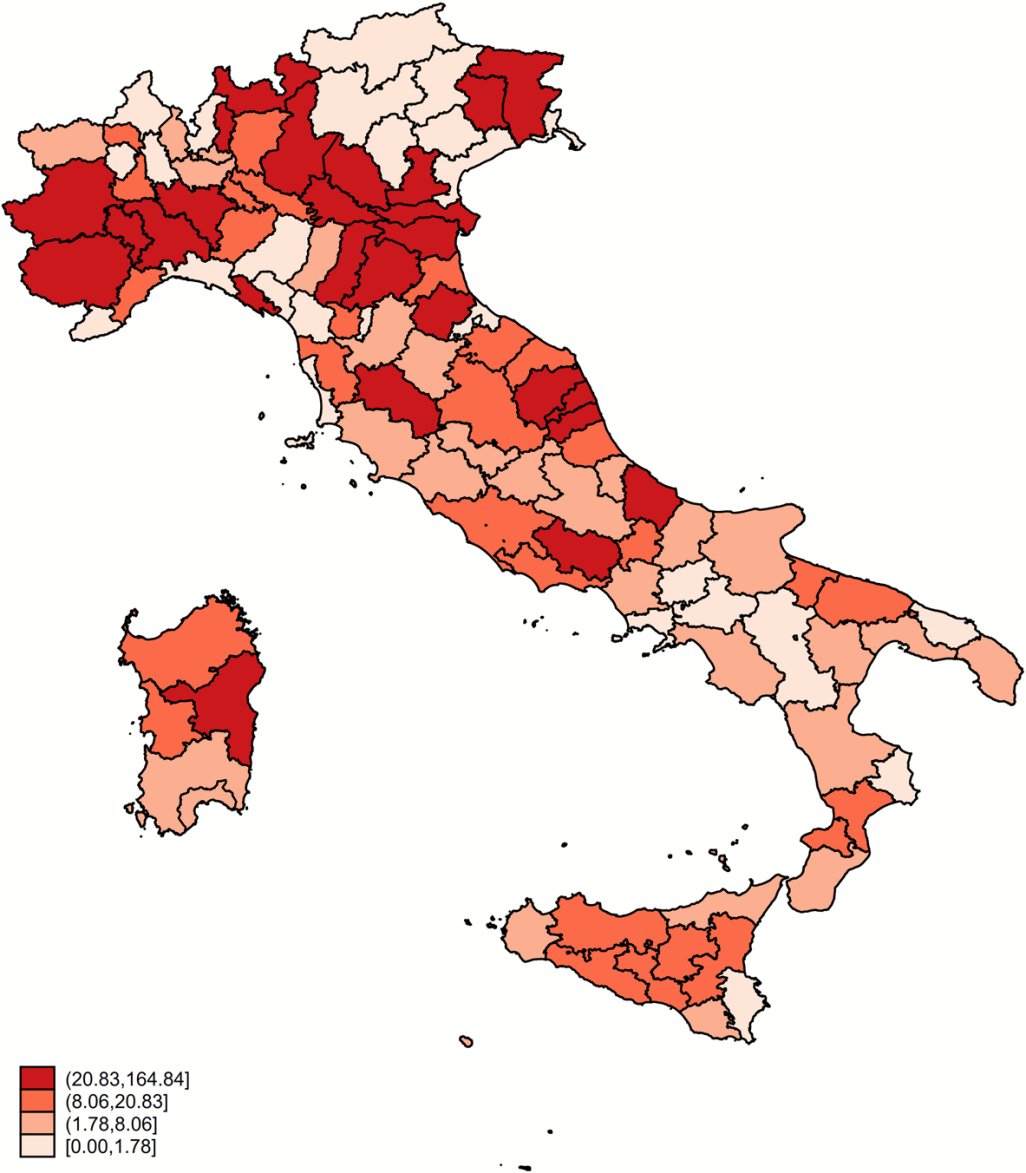
**Crude prevalence of scrapie (cases per 10 000 tests) by quartile at provincial level over the 2002–2022 period**.

Over the period 2002–2022, the provincial crude prevalence shows values of positive tested sheep ranging from 0 to 164.84 per 10 000 tests performed, with a median value of 8 while the inter-quartile range is 1.78–20.83. Values above the 90^th^ percentile are found in North and central Italy, in the provinces of Ferrara, Mantua and Pavia (Figure 2).

**Figure 3:**
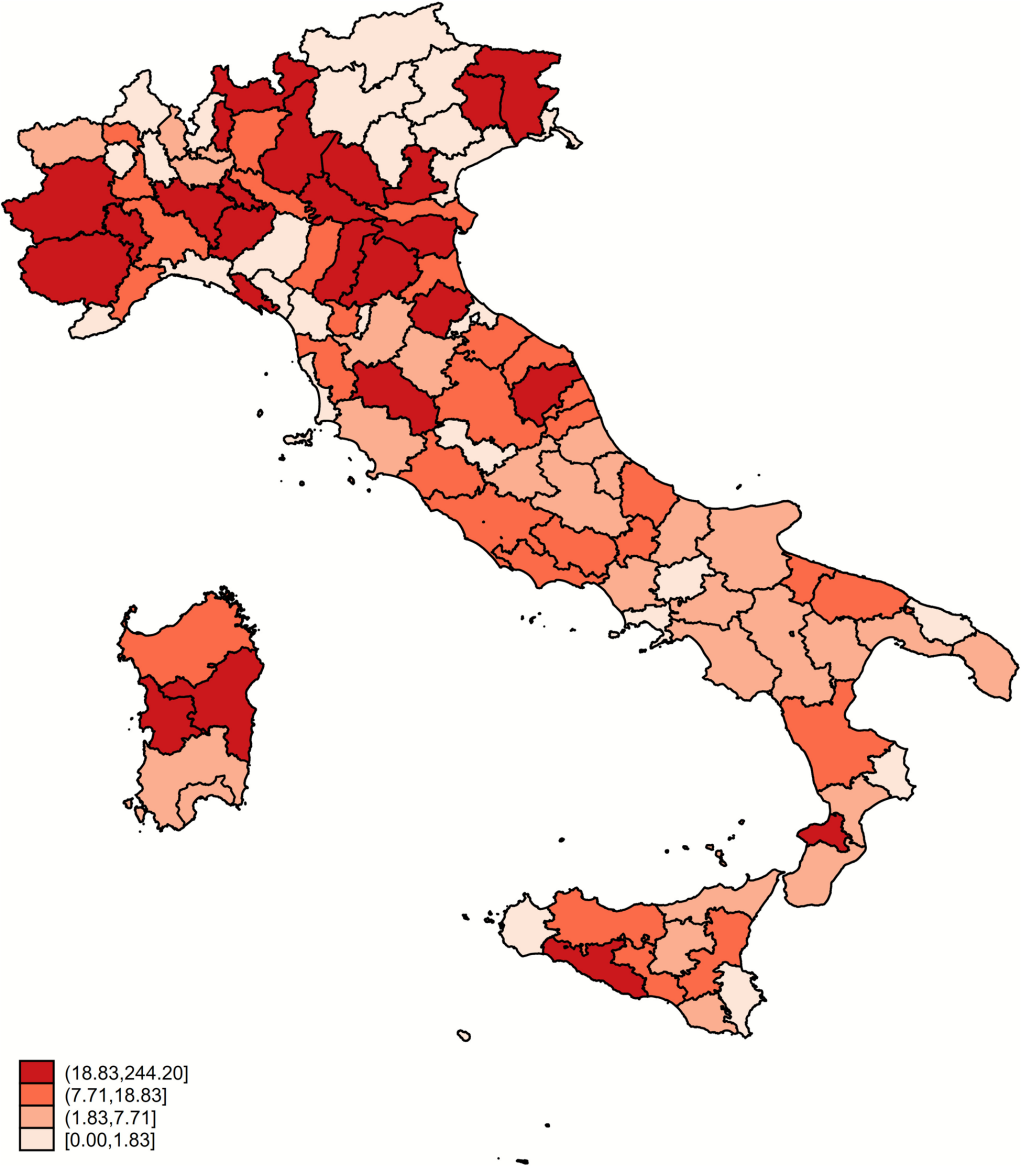
**Stream- and age class-adjusted prevalence of scrapie (cases per 10 000 tests) by quartile at provincial level over the 2002–2022 period**.

The provincial adjusted prevalence shows values ranging from 0 to 244.20 per 10 000 tests performed, with a median value of 7.71 and an inter-quartile range of 1.83–18.83 per 10 000. Values above the 90^th^ percentile are found in Northern Italy, in the provinces of Lodi and Pavia (Figure 3).

Scrapie shows considerable spatial heterogeneity, with higher prevalence in areas of Northern Italy and Sardinia, with a similar distribution (pattern) between crude and stream- and age-adjusted prevalence.

The total number of active surveillance tests during the period 2002–2022 was 569 084, including 759 positive tests (animals tested positive at rapid test in the frame of active surveillance) from 746 outbreaks (positive herds, where one or more index cases are detected). The total number of animals tested increased significantly over the years, peaking in 2007 with 81 155 tests. Macro-region 4 consistently had the highest number of animals tested in most years, followed by macro-region 3 and 6. The cumulative totals for macro-region 4 (152 038) and macro-region 6 (138 886) are the highest of all macro-regions and contribute significantly to the total of 569 084 tests over 21 years (Table 1). Scrapie prevalence by macro-region was apparently higher in age class 18–47 months, whereas the overall prevalence was higher in the category over 47 months of age (Table 2).

**Table 2:** **Scrapie surveillance results in sheep by age class and macro-region**

Macro-region	Age class (month)	*N* positives	*N* tested	% of tested by age class	% (positives/tested)
1	18–47	66	27 302	4.80	0.24
1	>47	23	12 668	2.23	0.18
2	18–47	36	27 515	4.83	0.13
2	>47	16	14 132	2.48	0.11
3	18–47	96	85 585	15.04	0.11
3	>47	79	47 017	8.26	0.17
4	18–47	70	113 002	19.86	0.06
4	>47	24	39 036	6.86	0.06
5	18–47	35	37 215	6.54	0.09
5	>47	30	26 726	4.70	0.11
6	18–47	158	102 515	18.01	0.15
6	>47	126	36 371	6.39	0.35
Total		759	569 084	100	0.13

The table shows the number and the proportion of positive cases among tested animals. Macro-regions are: (1) North-West, (2) North-East, (3) Central, (4) Southern Italy, (5) Sicily, and (6) Sardinia.

A mixed-effects Poisson model (model 1) was used to test the hypothesis of an overall temporal trend over the period 2005–2022. The model, which included macro-region as a random effect and year (as a continuous variable), age class and surveillance stream as covariates, identified no temporal trend (PR = 0.97, 95% CI 0.94–1.01). However, a higher risk was associated with fallen stock (PR = 3.92, 95% CI 2.27–6.73) and with animals over 47 months old (PR = 1.49, 95% CI 1.08–2.05).

The results of the analysis of the change point show that for the entire country there is a 95% probability that a change point lies within 2015 and 2020 (Mean 2018, Std. dev. 0.99, MCSE 0.06, Median 2018 credibility interval 2015–2020) (Table 3).

**Table 3:** **Results of the change point regression analysis**

	Equal-tailed				
	Mean	Std. dev	MCSE	Median	[95% cred. interval]
Sardinia	2013	0.61	0.02	2013	2012–2015
Italy except Sardinia	2018	0.83	0.04	2018	2017–2020
Italy	2018	0.99	0.06	2018	2015–2020

Data are presented by geographical area with mean, standard deviation, Monte Carlo Standard Error, median and credibility interval of the posterior distribution for the change point year.

Considering separately the two periods of enforcement of the NGSP (2005–2015) and of its amendment (2016–2022), the results of the models fitted in the first and in the second periods change: in the first case, there is an increasing trend from 2005 to 2015 (PR = 1.09, 95% CI 1.04–1.15), whereas in the second period (2016–2022) it is evident a decreasing temporal trend (PR = 0.88, 95% CI 0.83–0.93) (Table 4).

**Table 4:** **Results of the Poisson model used to detect trends in the prevalence of scrapie in sheep by macro-region and period (2005–2015 and 2016–2022)**

Model 1							
Poisson regression model							
Based on modelling of standardized prevalence ratio (PR)							
2005–2015				2016–2022			
	PR	Trend	95% CI		PR	Trend	95% CI
Year	1.09	Pos	1.04–1.15	Year	0.88	Neg	0.83–0.93
Route	5.50		2.71–11.09	Route	1.13		0.71–1.78
Age class	1.43		1.01–2.03	Age class	2.09		1.17–3.74
Macro-region	0.23		0.09–0.58	Macro-region	0.17		0.07–0.42

2005–2015				2016–2022			
Macro-region	PR	Trend	95% CI	Macro-region	PR	Trend	95% CI
North-West	1.14	Pos	1.03–1.26	North-West	0.92	–	0.77–1.10
North-East	1.11	–	0.97–1.26	North-East	0.9	–	0.72–1.14
Central Italy	1.01	–	0.95–1.08	Central Italy	0.83	neg	0.71–0.98
Southern Italy	1.09	Pos	1.02–1.17	Southern Italy	0.9	–	0.73–1.12
Sicily	1.35	Pos	1.22–1.50	Sicily	0.71	neg	0.53–0.95
Sardinia	1.06	–	1.00–1.14	Sardinia	0.92	–	0.80–1.05
Italy, except Sardinia	1.08	pos	1.04–1.13	Italy, except Sardinia	0.84	neg	0.78–0.90
Italy	1.06	pos	1.03–1.10	Italy	0.89	neg	0.83–0.97

**Figure 4:**
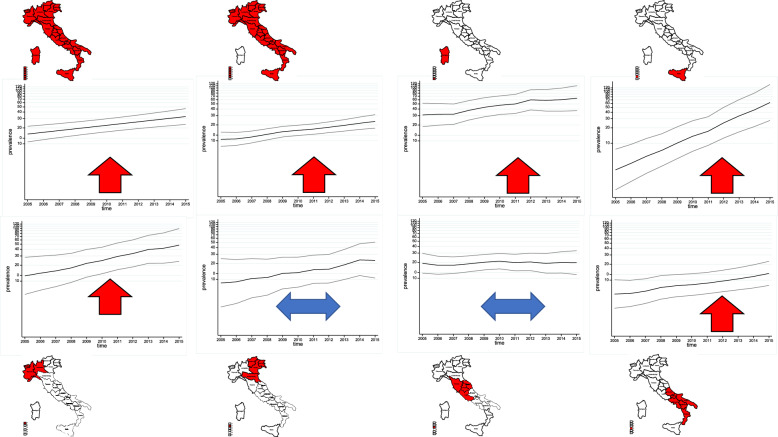
**Stream- and age class-adjusted annual prevalence rates by macro-region through Poisson regression models: linear trends calculated by macro-region and period (2005–2015)**.

**Figure 5:**
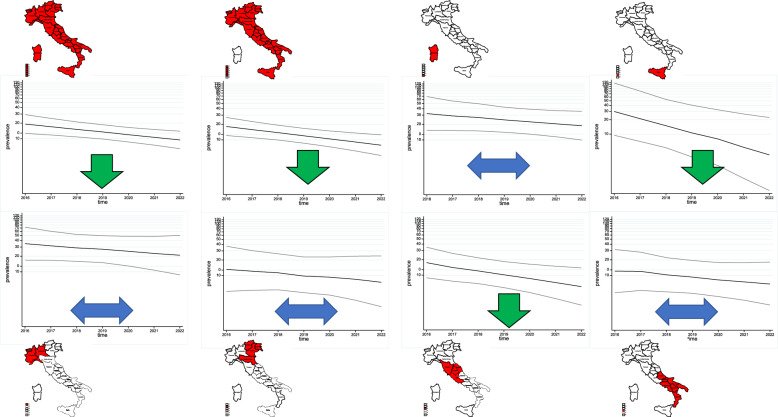
**Stream and age class-adjusted annual prevalence rates by macro-region through Poisson regression models: linear trends calculated by macro-region and period (2016–2022)**.

When the model was also fitted for each macro-region, the adjusted prevalence ratio varied between 0.71 and 1.35, showing an increase or decrease between the two periods depending on the macro-region, as shown in Table 4. The results of the Poisson regression models by macro-region are also summarised graphically in Figures 4 and 5. In period 1 (2005–2015), the adjusted prevalence rate ratio increases in all macro-regions except Central Italy and North-East Italy, whereas in period 2 (2016–2022) it shows an apparent decrease in some macro-regions except North-West Italy, North-East Italy, South Italy and Sardinia.

When the change-point analysis was applied separately to Sardinia and to the rest of Italy (excluding Sardinia), the estimated change point occurred between 2012 and 2015 in Sardinia and between 2017 and 2020 in the rest of the country. Detailed summary statistics are provided in Table 3.

### Genotyping

The total number of genotyped animals over the whole period was 813 307, of which 222 535 were susceptible, i.e. 27.36%, with the overall proportion per macro-region varying from 26% in Sardinia to 49% in North-East Italy.

In general, the number of genotyped animals increased over the period, especially after the introduction of the NGSP and subsequent amendments, while the number of susceptible animals decreased in all macro-regions and mainly in Sardinia, where the introduction of the 2009 regional plan led to an increase in genotyping activity.

**Figure 6:**
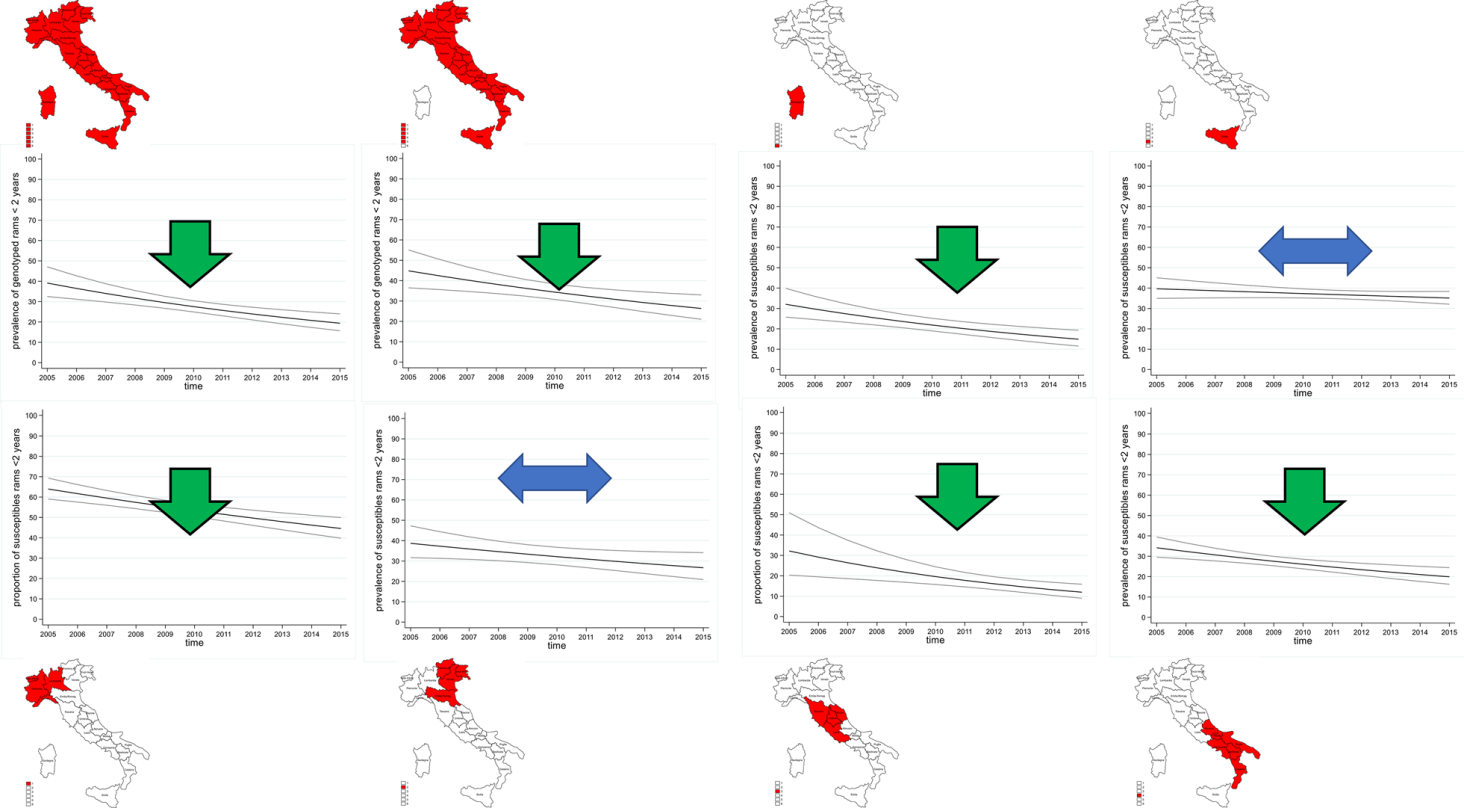
**Temporal trend of the proportion of susceptible rams among the youngest breeding rams (< 2 years) in the first period (2005–2015)**. Line shows linear time trend, outer lines show 95% confidence interval.

**Figure 7:**
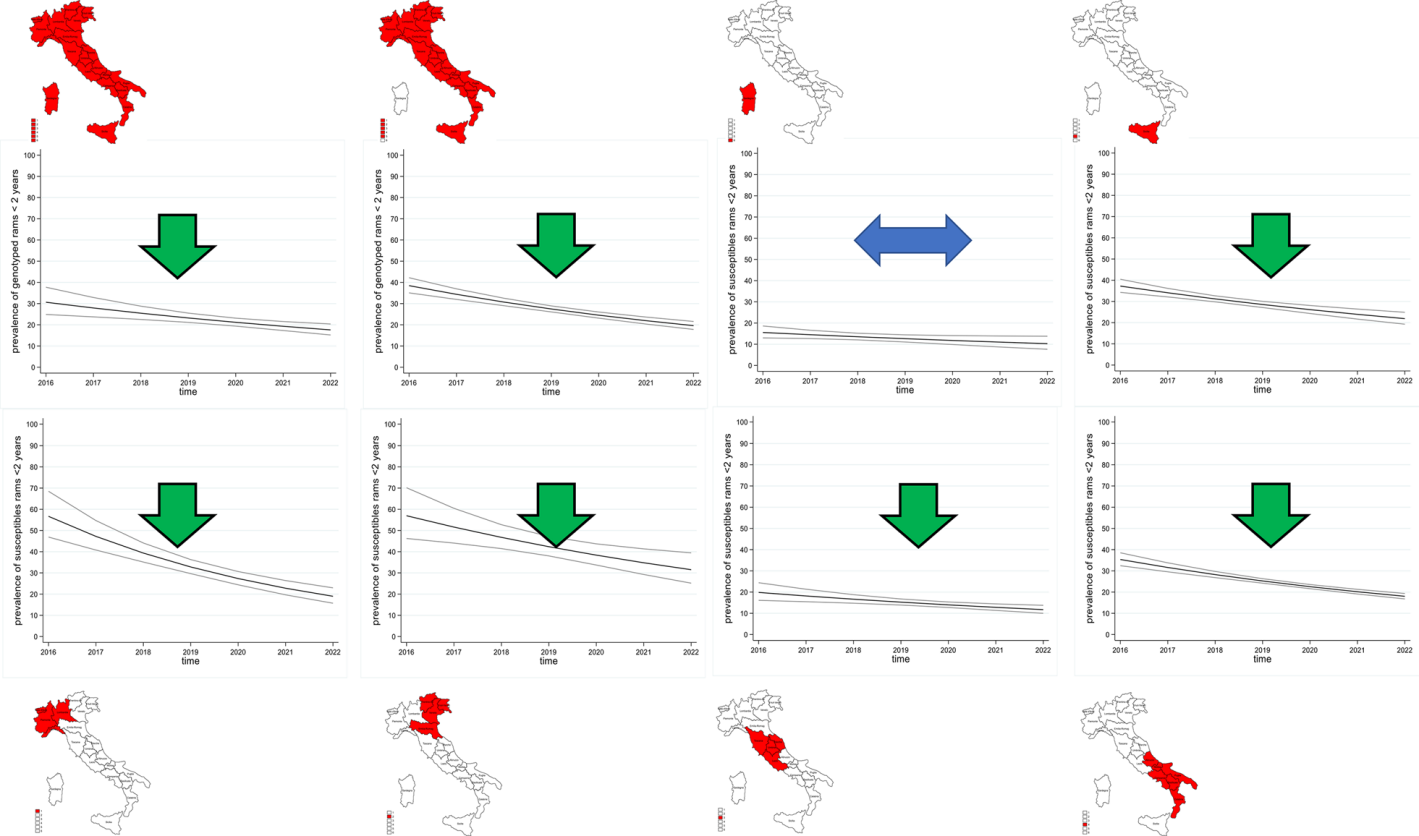
**Temporal trend of the proportion of susceptible rams among the youngest breeding rams (< 2 years) in the second period (2016–2022)**. Line shows linear time trend, outer lines show 95% confidence interval.

Figures 6 and 7 show the temporal trend of the proportion of susceptible rams aged less than 2 years in the entire country and by macro-region over period 1 and period 2, respectively. Over the entire period, at national level the proportion ratio of susceptible rams decreased by an average of 3% per year (PS 0.97, 95% CI 0.96–0.98). The decrease was more pronounced in the first period (PS 0.92, 95% CI 0.89–0.96) than in the second one (PS 0.94, 95% CI 0.89–0.98) (model 2), with an average of 8% and 6% per year, respectively (Table 5).

**Table 5:** **Results of the Poisson model used to detect trends in the proportion of susceptible sheep by macro-region and period (2005–2015 & 2016–2022)**

Model 2							
Poisson regression model							
Based on modelling of proportion of susceptible rams < 2 years old							
2005–2015				2016–2022			
Macro-region	PR	trend	95% CI	Macro-region	PR	trend	95% CI
North-West	0.96	neg	0.95–0.98	North-West	0.83	neg	0.79–0.88
North-East	0.96	–	0.93–1.00	North-East	0.91	neg	0.85–0.96
Central Italy	0.91	neg	0.85–0.96	Central Italy	0.92	neg	0.87–0.97
Southern Italy	0.95	neg	0.92–0.99	Southern Italy	0.91	neg	0.88–0.93
Sicily	0.99	–	0.97–1.00	Sicily	0.92	neg	0.89–0.94
Sardinia	0.93	neg	0.89–0.97	Sardinia	0.93	–	0.87–1.00
Italy, except Sardinia	0.95	neg	0.91–0.98	Italy, except Sardinia	0.89	neg	0.87–0.92
Italy	0.92	neg	0.89–0.96	Italy	0.94	neg	0.89–0.98

The results of the mixed Poisson regression model with province as a random effect (model 3) indicate that the scrapie adjusted prevalence rate ratio increases 2.16-fold (95% CI 1.47–3.18) when comparing the category with the lowest proportion of susceptible rams with the others (Table 6).

**Table 6:** **Results of the Poisson mixed regression model**

Model 3		
Poisson mixed regression model		
Based on modelling of standardized prevalence ratio		
Effects	PR	95%CI
Categorization of the proportion of susceptible animals (0–22% as referent; > 22%) (fixed)	2.16	1.47–3.18
Province (random)	0.80	0.48–1.32

The model was used to detect the association between the proportion of susceptible rams and scrapie prevalence accounting for province random effect. The categorisation of the proportion of susceptible rams is based on quartiles, with the result that it is classified as a dichotomous variable.

**Figure 8:**
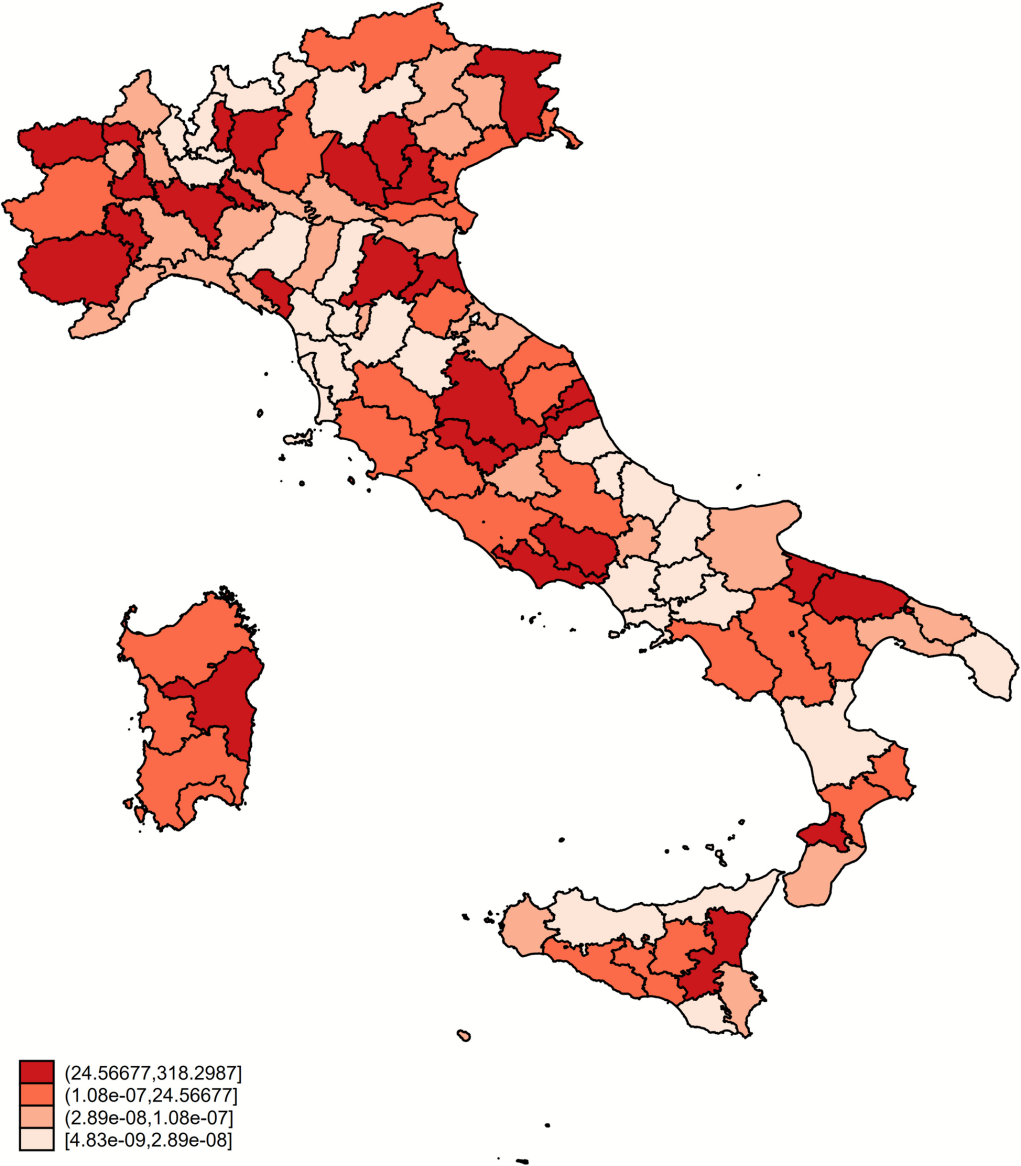
**Age class and stream adjusted prevalence at provincial level in the last 5 years (2018–2022).**

**Figure 9:**
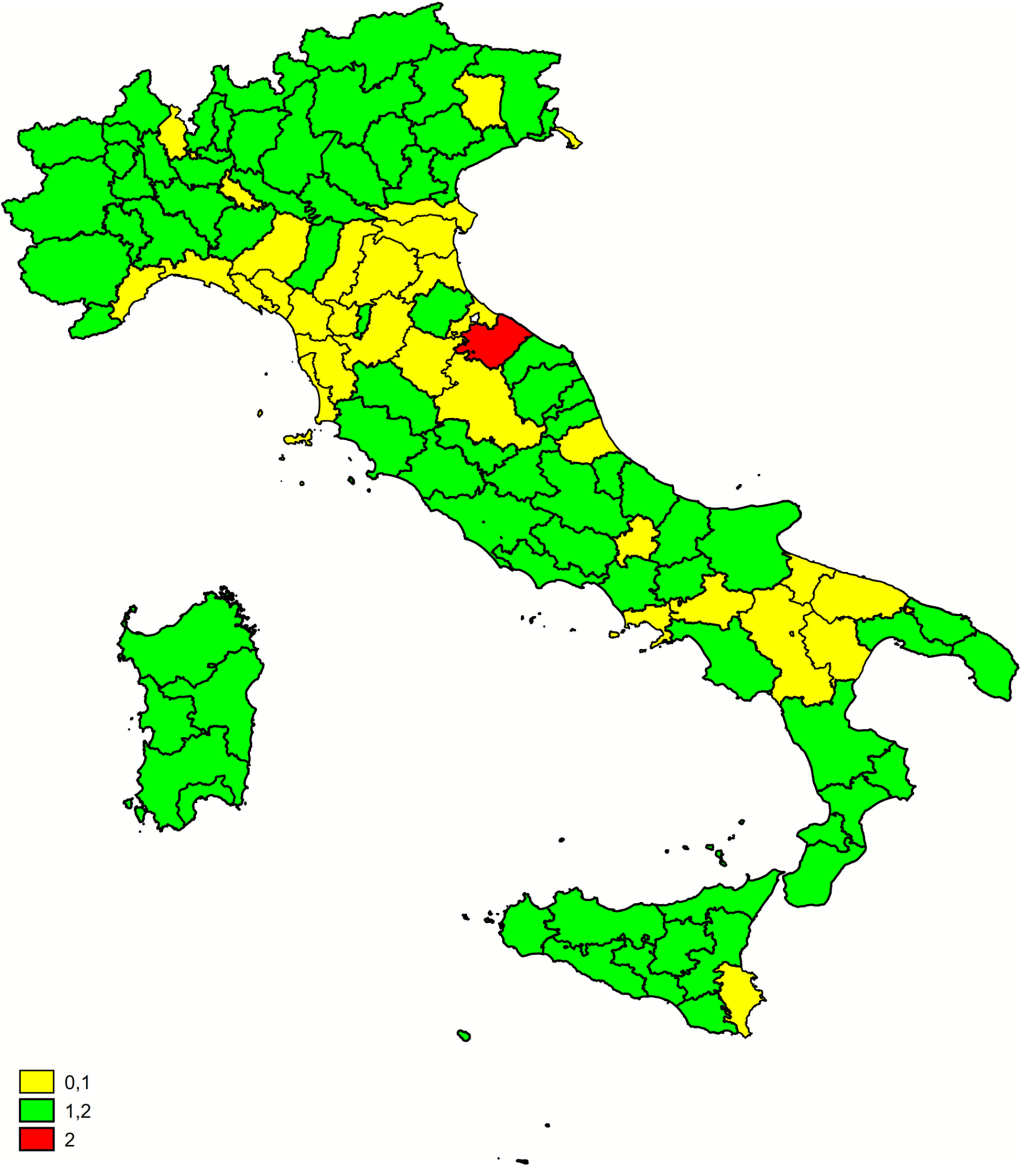
**Distribution of temporal trend of susceptible animals at province level (2016–2022)**. Red: increasing trend (PS > 1). Green: decreasing trend (PS < 1). Yellow: no trend (PS = 1).

Figures 8 and 9 show the output of models 4 and 5: in particular, the first figure illustrates the geographical distribution of the adjusted prevalence for the period 2018–2022, while the second figure shows the trend in the proportion of susceptible animals between 2016 and 2022.

By comparing the adjusted prevalence distribution with the proportion of susceptible animals, it is possible to identify areas where scrapie continues to significantly affect the sheep population despite the implementation of the NGSP. Notably, this includes regions in North-West and North-East Italy, as well as some areas in the South and in Sardinia.

## Discussion

The main result of this study is that the enforcement of a national breeding plan (NGSP), i.e. an intervention to combat scrapie in the Italian sheep population based on genetic selection of resistant animals, resulted in a decreasing trend in the prevalence of the disease in our country.

In general, a clear heterogeneity in the geographical distribution of the disease over the whole study period was evident even after adjusting the occurrence estimates on the level of the surveillance sensitivity applied. The decreasing trend varied significantly at national and macro-regional level, particularly when comparing two periods when distinct intervention plans have been launched and applied.

The first NGSP initially targeted high genetic merit flocks (HGMFs); the second NGSP modified the strategy and extended compulsory application to commercial flocks, irrespective of breed, production and commercial orientation. The effects of these changes are evident in our macro-regional trend analysis. In the first period there was an increase in prevalence in all the macro-regions except in a couple of them (i.e. Northeastern and Central Italy), while in the second period there was a decrease in Sicily and Central Italy.

Sardinia provides a key example of how regional differences in implementing the NGSP played a role: an ad hoc intervention in 2009 anticipated most of the provisions of the second NGSP and the change point regression analysis confirms a clear difference in outcomes between Sardinia and the rest of the country.

The effectiveness of the NGSP is further documented by the observed increase in genotyping activity (i.e. in the number of animals genotyped) after each modification of the plan, although in the second period the additional effort was less evident, especially in Sardinia. This increase was paralleled by the change in the frequencies of genotypes known to be associated with susceptibility to the disease. In this regard, the 2014 EFSA Opinion [35], which evaluated the effectiveness of the Italian breeding plan before 2015, provides critical context. Using a deterministic simulation model, EFSA concluded that the genetic selection plan produced results in high genetic merit flocks (HGMFs), but the dissemination of resistant genotypes was limited owing to poor national coverage and regional differences. The case of Sardinia, which contains half of the national sheep flock, illustrates this. Initially, significant efforts were made to implement the genetic selection plan and preserve production traits [36]. The plan was implemented in two stages: first, genotyping and selection of rams were mainly applied in HGMF to increase the availability of ARR carrier rams while preserving production traits. Despite these efforts, the HGMF animal nucleus was too small to supply commercial breeding farms with resistant males; the second step involved extending genotyping to the entire population and making the use of ARR rams mandatory.

EFSA’s assessment revealed that the Italian NGSP was characterised by the poor dissemination of resistant genetics, which resulted in a limited or delayed impact on the general sheep population. While the programme had some effect within high genetic merit flocks (HGMFs), its influence on commercial flocks was unclear. This was primarily because HGMFs constitute only around 7% of the entire sheep population, meaning they were unable to replace rams in commercial flocks effectively. The EFSA opinion concluded that the impact of the NGSP on the wider population was unclear and that a long observation period was required. Our study, however, shows that, over time thanks to the implementation of the second NGSP, animal genotyping data has increased paralleled by the proportion of resistant genotypes in the young male population (i.e. the target of the selective intervention), with a significant trend in nearly all macro-regions. This is encouraging, particularly in areas where disease outbreaks still occur.

Despite these positive trends, our study has certain limitations that warrant consideration. A key one is the incomplete consideration of sheep breed. It is well-established that breed composition is a relevant factor influencing scrapie susceptibility and the effectiveness of genetic selection programmes. Unfortunately, systematic information on breed was unavailable, as breed is not a mandatory data field in the Italian scrapie surveillance programs. This lack of detailed breed data restricts our ability to analyse the interaction between breed, genotype distribution and disease dynamics at the population level, and the study may overlook important heterogeneities in susceptibility and control outcomes across regions or flocks. Furthermore, we relied on the reduction of susceptible animals as a proxy for plan’s effectiveness; while a valid measure, it may not fully capture the complexity of scrapie control which is also influenced by factors such as incomplete adherence to mating recommendations, regional variations in adoption and differences in herd management practices.

As of 2023, Italy remains one of the few European countries alongside Greece, Romania and Spain that continues to report cases of classical scrapie (CS) in sheep. This contrasts with other EU member states, such as France, the UK, Ireland, the Netherlands and Slovenia, where statistically significant decreasing trends in CS prevalence have been observed (EFSA, 2023). This variability in outcomes likely reflects differences in farming systems, genetic resistance profiles and control measures. France, for example, implemented enhanced surveillance and comprehensive genetic selection strategies, including exhaustive testing and systematic culling of susceptible genotypes, which has led to more effective outbreak control. In contrast, Italy’s sheep industry is characterised by high breed diversity and regional variation in allele frequencies, with some areas showing a predominance of susceptible genotypes. Furthermore, extensive production systems, high flock density and animal movements for mating or sale purposes present additional challenges to control efforts.

The persistence of scrapie in Italy, despite the evolution of EU regulations allowing for more flexible, genotype-based interventions, suggests a need for more harmonised and rigorous implementation of these strategies. Our provincial-level analyses show that there are some critical areas where further investigation is needed. Understanding the spatial heterogeneity of the disease and identifying local risk factors will be crucial for improving the effectiveness of the national scrapie control strategy and ensuring that the dissemination of resistant alleles is no longer limited.

## Supplementary Information

**Additional file 1.** **Genotyped sheep by year, macro-region, and age group, showing the number and percentage of sheep genotyped for** ***PRNP*** **that were under and over two years old**. Macro-regions are: (1) North-West, (2) North-East, (3) Central, (4) Southern Italy, (5) Sicily, and (6) Sardinia.

## Data Availability

Data provided in the framework of TSE surveillance plans.

## Abbreviations

TSE: transmissible spongiform encephalopathies
NGSP: National Genetic Selection Plan
PR: prevalence ratio
PS: proportion of susceptible animals
